# Deep brain stimulation in the globus pallidus alleviates motor activity defects and abnormal electrical activities of the parafascicular nucleus in parkinsonian rats

**DOI:** 10.3389/fnagi.2022.1020321

**Published:** 2022-09-28

**Authors:** Jinlu Xie, Zheng Chen, Tingting He, Hengya Zhu, Tingyu Chen, Chongbin Liu, Xuyan Fu, Hong Shen, Tao Li

**Affiliations:** ^1^Laboratory of Vector Biology and Pathogen Control of Zhejiang Province, School of Medicine, Huzhou University, Huzhou, China; ^2^Key Laboratory of Animal Resistance of Shandong Province, College of Life Sciences, Shandong Normal University, Jinan, China; ^3^Key Laboratory of Biomedical Engineering of Ministry of Education, College of Biomedical Engineering and Instrument Science, Zhejiang University, Hangzhou, China; ^4^Department of Neurology, Huzhou Central Hospital, Affiliated Center Hospital of Huzhou University, Huzhou, China; ^5^Department of Physical Education, Kyungnam University, Changwon, South Korea

**Keywords:** Parkinson’s disease, deep brain stimulation, globus pallidus, parafascicular nucleus, therapy

## Abstract

Deep brain stimulation (DBS) is an effective treatment for Parkinson’s disease (PD). The most common sites targeted for DBS in PD are the globus pallidus internal (GPi) and subthalamic nucleus (STN). However, STN-DBS and GPi-DBS have limited improvement in some symptoms and even aggravate disease symptoms. Therefore, discovering new targets is more helpful for treating refractory symptoms of PD. Therefore, our study selected a new brain region, the lateral globus pallidus (GP), as the target of DBS, and the study found that GP-DBS can improve motor symptoms. It has been reported that the thalamic parafascicular (PF) nucleus is strongly related to PD pathology. Moreover, the PF nucleus and GP have very close direct and indirect fiber connections. However, whether GP-DBS can change the activity of the PF remains unclear. Therefore, in this study, we monitored the activity changes in the PF nucleus in PD rats during a quiet awake state after GP-DBS. We found that GP-DBS could reverse the electrical activity of the PF nucleus in PD model rats, including the discharge pattern of the neurons and the local field potential (0.7–12 and 12–70 Hz). Based on the results mentioned above, PF activity in PD model rats could be changed by GP-DBS. Thus, the normalization of PF neuronal activity may be a potential mechanism for GP-DBS in the treatment of PD; these findings lay the foundation for PD treatment strategies.

## Introduction

Parkinson’s disease (PD) is a chronic progressive neurodegenerative disorder with motor dysfunction, manifesting bradykinesia, rigidity, resting tremor, loss of postural reflexes, postural instability, and gait disturbances (Armstrong and Okun, 2020). The current clinical treatment of PD mainly involves levodopa-based drug therapy and deep brain stimulation (DBS). However, long-term drug treatment is accompanied by side effects, such as end-dose reactions and dyskinesias. Thus, DBS is an effective treatment for alleviating symptoms in PD, especially for individuals in advanced stages of PD (Benabid et al., 2009; Udupa and Chen, 2015; Qiu et al., 2016; Allert et al., 2018; Kim et al., 2018; Vizcarra et al., 2019; Armstrong and Okun, 2020; Marsili et al., 2020).

For a long time, the subthalamic nucleus (STN) and globus pallidus internus (GPi) have been the preferred targets for DBS in PD treatment (Kleiner-Fisman et al., 2006; Amara et al., 2012; Schuepbach et al., 2013; Jz et al., 2020). However, Conventional treatments for PD such as STN-DBS or GPi-DBS are often minimally effective or can even worsen some symptoms, such as gait freezing, postural instability balance, and cognitive (Alvarez et al., 2005; Stefani et al., 2007; Heo et al., 2008; Okun et al., 2009). It is urgent to find novel targets for DBS in the treatment of PD. The globus pallidus (GP, equivalent to the external GP in primates) has been recently promoted and investigated (Vitek et al., 2004, 2012; Johnson et al., 2012; Mastro et al., 2017; Crompe et al., 2020). The GP is a critical node in the indirect pathway of the basal ganglia. It projects to the STN, GPi, substantia nigra pars reticular, and motor cortex and receives inputs from the STN, striatum, substantia nigra pars compacta, and parafascicular nucleus (PF) (Sadikot and Rymar, 2009; Mallet et al., 2012; Ellens and Leventhal, 2013). An increasing number of electrophysiological, anatomical, and molecular studies have shown that the GP comprises heterogeneous populations of neurons participating in different circuits (Bolam et al., 2000; Sato et al., 2000; Kita and Kita, 2001; Kita et al., 2007; Nóbrega-Pereira et al., 2010; Benhamou et al., 2012; Mastro et al., 2014). Notably, GP-DBS not only improves the motor and non-motor symptoms in PD but also modulates the neuronal activity of the prominent nuclei of basal ganglia-thalamocortical circuits, including the thalamus, GP, and primary motor cortex (Ligot et al., 2011; Vitek et al., 2012; Mastro et al., 2017). Thus, in this study we selected the GP as the DBS target.

The striatum is the primary input site of the basal ganglia and affects the motor symptoms of PD through the direct and indirect pathways of the basal ganglia. PF builds a close relationship with the basal ganglia through the glutamatergic output to the dorsolateral striatum (Smith et al., 2014). More than 30% of the neurons in the PF were lost in PD patients and PD animal models, but neighboring thalamic nuclei remain intact (Henderson et al., 2000; Halliday, 2009). In addition, there were changes in the activity of the remaining neurons in the PF of patients or rat models of PD (Parr-Brownlie et al., 2009; Kempf et al., 2010). A study by our group showed that the electrophysiological activity of the PF in PD rats was significantly altered during a quiet awake state (Wang et al., 2016). The PF is closely related to PD and tightly associates with the GP nucleus. Recent studies have shown that there was a type of behavior-related neuron in the GP projecting to the PF, and these neurons could modulate the thalamostriatal pathway (Mastro et al., 2014; Lilascharoen et al., 2021). GP-DBS are most likely to improve the PF electrophysiological activities of PD model rats. However, how the electrical activity of the PF nucleus changes after GP-DBS is still unclear.

In this study, we selected the GP nucleus as the new target for DBS in PD models of rats. PD rats were treated with high-frequency stimulation on GP for 2 weeks. We performed the behavioral test to determine the effects of GP-DBS and found GP-DBS could improve execution disorders in both the vibrissae-evoked forelimb placing and the adjusting-steps tests. After confirming the therapeutic effect of GP-DBS on PD rats, we collected the electrical signals of PF in rats in a quiet awake state. Then we analyzed the improvement of PF electrical activity, including the spikes and local field potentials (LFPs). Thus, it further revealed the possible electrophysiological mechanism of GP-DBS in PD treatment and provided a theoretical basis for selecting a DBS treatment strategy for clinical PD.

## Materials and methods

### Animals

Wistar rats weighing 280–310 g were obtained from the Animal Center of Shandong University, China. These rats were housed in cages at room temperature (22 ± 1°C) with 14/10 h light/dark cycles and provided with food and water *ad libitum*. According to the National Institutes of Health guidelines for the Care and Use of Laboratory Animals, all animal experiments were approved by the Animal Ethics Committee of Shandong Normal University. The rats were categorized into the control group, lesioned group (6-OHDA-lesioned rats), and the DBS treatment group (6-OHDA-lesioned rats treated with DBS).

### Unilateral lesion of the nigrostriatal pathway and electrode implantation

The rats were anesthetized with 4% chloral hydrate (400 mg/kg, i.p.). Dopaminergic neurons were lesioned by injecting 3 μL 6-OHDA (4 μg/μL) into the right medial forebrain bundle (anterior [A]: –1.72 mm; lateral [L]: 2.13 mm; ventral [V]: –8.5/-8.7 mm from bregma) following the brain atlas of Paxinos and Watson (2007). The control rats received only the vehicle (0.02% ascorbic acid in physiological saline). The operation was described in our previous study (Wang et al., 2016). After lesion surgery, recording electrodes composed of 16 nickel-chromium Teflon-insulated microwires were stereotaxically implanted into the PF of the rats in all groups (coordinates for the PF: A: –4.2 mm; L: 1.2 mm; V: –5.8 mm). For all the rats, stainless-steel electrodes with a diameter of 0.18 mm were simultaneously implanted unilaterally (ipsilateral to the 6-OHDA lesion) into the GP (A: –1.0 mm; L: 3.0 mm; V: –6.6 mm). Finally, the electrodes were fixed with dental cement on the rats’ skulls. After surgery, 0.5 ml (160 mg/kg, i.p.) of penicillin was administered to rats for 3 days to prevent infection.

### Evaluation of rat models of Parkinson’s disease

The extent of 6-OHDA lesions in rats in the lesioned and DBS treatment groups was assessed *via* an apomorphine (APO)-induced rotation test 1 week after lesion surgery. The rat rotations were counted for 30 min after APO (0.5 mg/kg, i.m.) injection. The rats that rotated over seven circles per minute by the contralateral limb and lasted over 30 min were considered successful models. Only these rats were used for behavioral tests and electrophysiological recordings. The above rats were randomly assigned to either the lesioned group (*n* = 7) or the DBS treatment group (*n* = 8).

### Stimulation

Deep brain stimulation was performed twice a day for 30 min each session on rats in the DBS treatment group, with an interval of 12 h between sessions. Stimulation pulses with a frequency of 150 Hz, an amplitude of 1.5 volts, and a width of 60 μs were produced by a multichannel amplification system (RM6280BD, Chengdu Instrument Factory, Chengdu, China).

### Electrophysiological data acquisition and analysis

Three weeks after the operation, the extracellular signals of the PF of all rats were recorded during a quiet awake state using a 16-channel OmniPlex D neural data acquisition system (Plexon Inc., Dallas, USA). Following the guidelines provided by Wang et al. (2016), the signals were split into spike and LFP activities by digitizing with sampling rates of 20 kHz and 1,000 Hz and bandpass filtering at 300–8,000 and 0.7–200 Hz, respectively. The raw spike signals were stored for further sorting analysis using Offline Sorter (Plexon Inc., Dallas, USA). The sorted signals of every neuron were then cut into six segments (6 × 10 s length) and analyzed with NeuroExplorer (Nex Technologies, USA). For each rat, 12 × 10 s of LFP data recorded in the PF were analyzed offline with LFP analysis software 2009^1^ in MATLAB 2010a (The MathWorks, USA). The spike signals were classified according to the characteristic values of waveforms and interspike interval (ISI) histograms. During a quiet awake state, 168 type I neurons were collected, 66 of which were from six control rats, 55 of which were from seven 6-OHDA-lesioned rats, and 47 of which were from eight DBS-treated rats. In addition, 95 type II neurons were collected, 36 of which were from control rats, 27 of which were from 6-OHDA-lesioned rats, and 32 of which were from DBS-treated rats. We then analyzed and compared the firing rate of neurons and the coefficient of variance (CV) of the spike patterns among neurons in the three groups.

The firing rate of spikes was defined as the counts of spikes divided by time. The CV value, which was calculated through ISI analysis, described the regularity of neuronal discharge. More irregular firing patterns were observed when the CV values were over 1; in contrast, more regular firing patterns appeared when the CV values were lower than 1 (Geng et al., 2016).

Local field potential activity was described by time-frequency spectrograms, power spectra density, and spectral decomposition. The bands of 0.7–12, 12–35, 35–70, and 70–200 Hz were analyzed. The total power percentage was calculated and compared among the three groups within each band range. The detailed parameter settings in this analysis were described in our previous study (Wang et al., 2015).

### Vibrissae-evoked forelimb placing test and adjusting-steps test

The effects of GP-DBS on the rats’ behavior were evaluated *via* vibrissae-evoked forelimb placement and adjusting-steps tests after the DBS treatments were completed (i.e., 3 weeks after surgery). In the vibrissae-evoked forelimb placing test, each rat’s torso, two hindlimbs, and healthy forelimbs were gently held, and the affected forelimbs were kept suspended (Schallert et al., 2002). The vibrissae were then brushed against the edge of a table to elicit a placing response in the aloft forelimb. Intact rats can instinctively place their forelimb upon any nearby surface they sense, whereas lesioned rats may fail in this test because of the ipsilateral side motor of lesioned deficits. For each test, ten trials were performed in each block for a total of ten blocks. Each successful practice was marked with one score, and the total score of each trial was averaged across all ten blocks. Finally, the mean score of each rat was used for statistical analysis.

An adjusting-steps test was performed to evaluate the forelimb akinesia of rats (Olsson et al., 1995; Winkler et al., 1999). Each rat was held almost vertically upside down by the torso, including two hindlimbs and one forelimb ipsilateral to the lesion side, and the other forelimb was kept down and allowed to touch a surface. The tip of the nose and the forelimb touching a surface were aligned with the start line for each rat. Then, the rats were slowly moved forward on the single free forelimb. The rats could only use their single free forelimb to regain their center of gravity in the process. The test results were quantified as the number of adjusting responses used to regain the center of gravity with a constant distance of 40 cm and a continuous 5 s. Each forelimb was independently examined, and the averaged value across ten times was used for statistical analysis.

### Histology and immunohistochemistry

**FIGURE 1:**
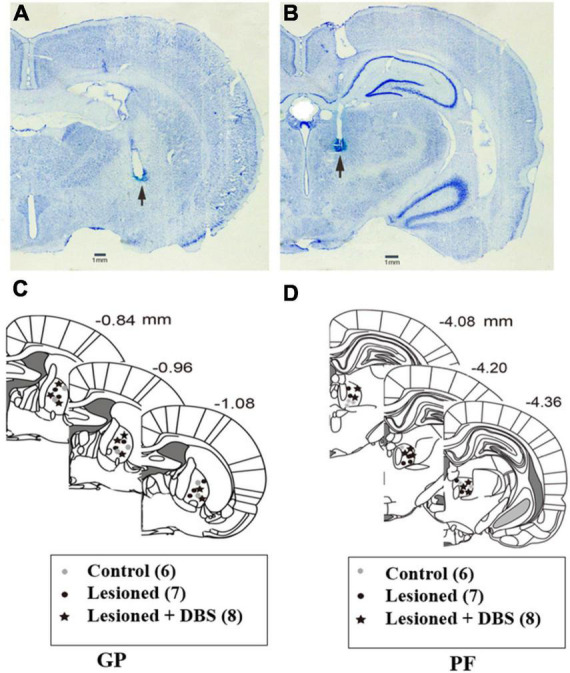
Two representative examples of neutral Nissl-stained coronal sections of a rat brain are pointing to the tip of a stimulating electrode in the GP **(A)** and the recording electrode in the PF **(B)**. Black arrows indicate the locations of the electrodes’ tips. Schematic reconstructions adapted from Paxinos and Watson (2007) represent the sites of the stimulating electrode tip in the GP **(C)** and the recording electrode tip in the PF **(D)**. Control: control rats (gray circles); Lesioned: 6-OHDA-lesioned rats (black circles); Lesioned + DBS: 6-OHDA-lesioned rats with DBS (stars). The number beside each schematic indicates the distance from bregma in the anteroposterior plane.

After all the experiments were completed, the rats were sacrificed by an overdose of 4% chloralhydrate (i.p.). A positive current of 1 mA was passed through the electrodes for 5 s (10 times) to mark the placement of the recording. Then, the rats were perfused with 0.1 M phosphate buffer solution (PBS), then followed by 4% paraformaldehyde in PBS containing 1% potassium ferricyanide. The rats’ brains were removed from the skull and then dehydrated in 30% sucrose in PBS. For structural verification, coronal sections (30 μm) encompassing the GP and PF were cut and stained with cresyl violet. Only the rats with correct recordings and stimulation electrode placements (Figures 1A–D) were used for data analysis.

To evaluate the survival of dopaminergic neurons in the substantia nigra pars compacta (SNc) of rats, we performed immunohistochemical staining of tyrosine hydroxylase (TH). Immunohistochemistry was performed as previously described (Wang et al., 2016). Mouse anti-tyrosine hydroxylase (ab6211; Abcam, Inc., Cambridge, UK) was used as the primary antibody for the immunohistochemical identification of TH; biotinylated secondary antibodies (Zymed Laboratories, Inc., CA, USA) and avidin horseradish peroxidase-diaminobenzidine system (DAB; Sigma Chemical, MO, USA) were used to detect the primary antibodies. After immunohistochemistry, TH-positive neurons in the SNc were counted, and the distance between the quantified sections was 120 μm (bregma −4.80 mm to −5.28 mm).

### Statistical analysis

Data were presented as the means ± standard error of the mean (SEM) and analyzed by SPSS 18 software (SPSS Inc., Chicago, USA). The student’s *t*-test was used to compare the number of TH immunoreactive neurons on the non-lesioned and the lesioned side of SNc of rats. The date of behavioral tests, the firing rate, the CV values, and each frequency band of LFP were analyzed separately by one-way ANOVA. In this study, ANOVA followed LSD *post-hoc* comparison when equal variances were assumed and Dunnett *post-hoc* comparison when equal variances were not assumed. The level of significance was set at *p* < 0.05.

## Results

### Behavioral effects of globus pallidus-deep brain stimulation

**FIGURE 2:**
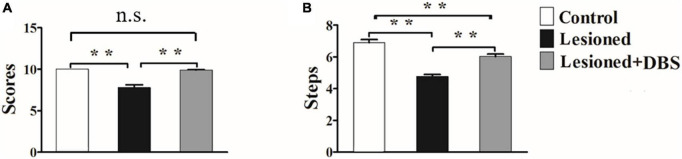
Results of the vibrissae-evoked forelimb placing test **(A)** and adjusting-steps test **(B)** in rats in the control group (*n* = 6), 6-OHDA-lesioned group (*n* = 7), and 6-OHDA-lesioned + DBS treatment (*n* = 8) group. **p < 0.01.

The analysis of variance (ANOVA) results for the vibrissae-evoked forelimb placing test showed a significant difference among rats in the three groups (*F*_(2,18)_ = 31.12, *p* < 0.01) (Figure 2A). Rats in the control group had higher scores than rats in the 6-OHDA-lesioned group (10.00 ± 0.00 vs. 7.77 ± 0.38; *p* < 0.01). Moreover, a significant difference was observed between rats in the 6-OHDA-lesioned and DBS treatment groups (7.77 ± 0.38 vs. 9.90 ± 0.06; *p* < 0.01). However, no significant difference was noted between rats in the control group and rats in the DBS treatment group. Therefore, the success score of rats in the DBS treatment group was almost reversed to normal.

The results of ANOVA for the adjusting-steps test are presented in Figure 2B. There was a certain difference in the number of the steps of the rats among the groups (*F*_(2,18)_ = 46.88, *p* < 0.01). The number of adjusting steps decreased in rats in the 6-OHDA-lesioned group compared with those in the control group (4.76 ± 0.13 vs. 6.9 ± 0.19; *p* < 0.01). Furthermore, the number of adjusting steps in rats in the DBS treatment group was significantly higher than that in rats in the 6-OHDA-lesioned group (6.03 ± 0.15 vs. 4.76 ± 0.13; *p* < 0.01) and was close to that in rats in the control group (6.03 ± 0.15 vs. 6.9 ± 0.19; *p* < 0.01).

The above results showed that the motor function of the injured forelimb of PD model rats was relieved after GP-DBS treatment.

### Effects of globus pallidus-deep brain stimulation on the spike activity in the parafascicular

**FIGURE 3:**
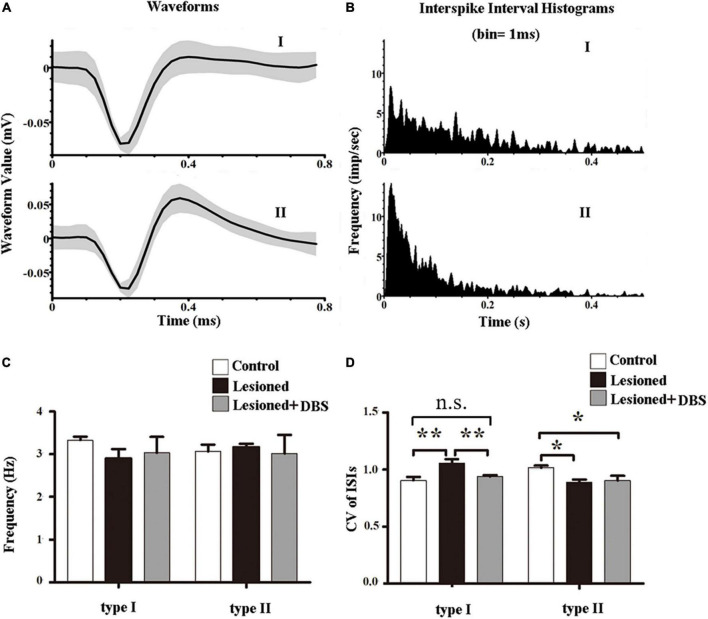
Effects of GP-DBS on the spike activity of PF. **(A)** Examples of waveforms of type I and type II neurons. Black lines represent averaged waveforms. **(B)** ISI histograms of type I and type II neurons. **(C)** Firing rates of the two types of neurons in the PF. **(D)** CVs of the ISIs for the two types of neurons in the PF. *p < 0.05, **p < 0.01.

Only data from rats with correct placement of the recording and stimulating electrodes were used for statistical analysis. We used offline sorter V4 software (OFS, Plexon Inc., USA) to perform spike sorting based on principle-component analysis (PCA). The neurons with cluster points in a 3-dimensional feature space were isolated in the PCA. After detailed computer analysis, two waveform types of neurons were shown. The distribution of the ISI histograms exhibited clear variations between the two subtypes. We categorized PF neurons into two types according to the electrophysiological characteristics: I and II (Figure 3A). The distribution of the ISI in the type I cells was characterized as relatively broad and random. In contrast, the distribution of the ISI in the type II cells was characterized as a positively skewed distribution with an initial sharp peak (Figure 3B). Analysis of each type of PF neuron indicated no differences in spike firing rates among rats in the three groups (type I: [*F*_(2,18)_ = 0.54, *p* = 0.59]; type II: [*F*_(2,18)_ = 0.06, *p* = 0.938]) (Figure 3C). These results showed that 6-OHDA lesions and GP-DBS had no significant difference in the firing rates of neurons in the PF.

However, the spike firing patterns were altered (Figure 3D). There were differences in the firing patterns of type I neurons in the PF of rats among the three groups (*F*_(2,18)_ = 8.68, *p* < 0.01). Furthermore, the CV values of the 6-OHDA-lesioned rats were significantly higher than those of both the control rats (1.06 ± 0.04 vs. 0.91 ± 0.03; *p* < 0.01) and DBS-treated rats (1.06 ± 0.04 vs. 0.94 ± 0.01; *p* < 0.01). No difference in CV value was observed between rats in the control group and rats in the DBS treatment group (0.91 ± 0.03 vs. 0.94 ± 0.01; *p* = 0.39). These results indicated that the firing pattern of type I neurons in the PF of 6-OHDA-lesioned rats was more irregular than that of control rats, and GP-DBS improved the condition. Also, the firing patterns of type II neurons in the PF were different among the three groups (*F*_(2,18)_ = 4.27, *p* < 0.05). The CV value of the 6-OHDA-lesioned rats was significantly lower than that of the control rats (0.89 ± 0.02 vs. 1.02 ± 0.02; *p* < 0.05). In contrast, the CV values of the DBS-treated rats were not significantly different from those of the 6-OHDA-lesioned rats (0.91 ± 0.04 vs. 0.89 ± 0.02; *p* = 0.73), indicating that GP-DBS could not improve changes in the firing patterns of the type II neurons in the PF of PD model rats.

### Effects of globus pallidus-deep brain stimulation treatment on local field potential in the parafascicular

**FIGURE 4:**
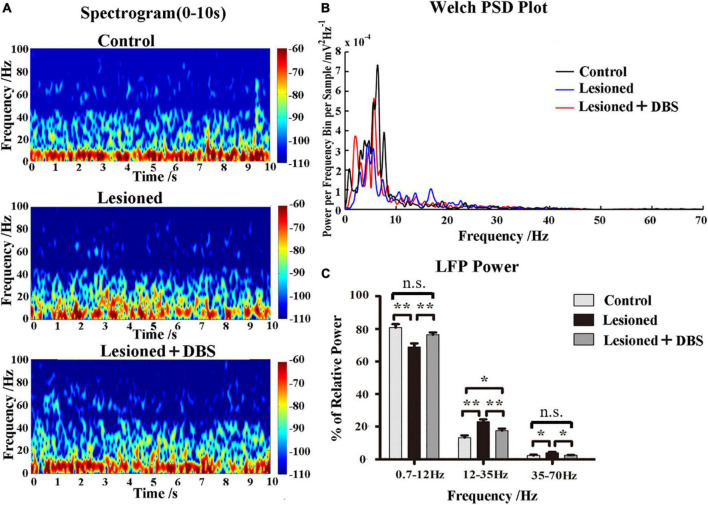
Effects of GP-DBS treatment on the local field potential (LFP) in the PF. **(A)** Time-frequency spectrograms of LFP powers in the PF from control, 6-OHDA-lesioned, and DBS-treated rats. **(B)** Welch estimation analysis of LFP powers in the PF from control, 6-OHDA-lesioned, and DBS-treated rats. **(C)** Relative LFP powers with a series of frequency ranges: 0.7–12, 12–35, and 35–70 Hz in control, 6-OHDA-lesioned, and DBS-treated rats. *p < 0.05, **p < 0.01.

The results of LFP data analysis showed that DBS could reverse the changes in neural activities caused by 6-OHDA. The LFP data during a quiet awake state were recorded from rats in the control (*n* = 6), 6-OHDA-lesioned (*n* = 7), and DBS treatment (*n* = 8) groups. The time-frequency spectrograms showed that the LFP powers in the PF of 6-OHDA-lesioned rats changed compared with those of the control rats and almost reversed to the level of the control rats after GP-DBS treatment (Figure 4A). Welch’s estimation of the LFP power spectra showed a similar result (Figure 4B). The LFP power of rats in the 6-OHDA-lesioned group was suppressed in the low-frequency bands but increased in the high-frequency bands. Notably, GP-DBS treatment reversed this phenomenon.

To further evaluate and quantify the effect of GP-DBS treatment on LFPs in the PF, we analyzed the relative power of four frequency bands (0.7–12, 12–35, 35–70, and 70–200 Hz) of rats in the three groups (Figure 4C). ANOVA results showed that the relative power changes in the three frequency bands of 0.7–12 (*F*_(2,18)_ = 9.277, *p* = 0.002), 12–35 (*F*_(2,18)_ = 12.631, *p* = 0.00), and 35–70 Hz (*F*_(2,18)_ = 5.12, *p* = 0.017) were significantly different, and there was no significant difference in the 70–200 Hz band (*F*_(2,18)_ = 0.088, *p* = 0.916). The *post-hoc* comparison showed that the 0.7–12 Hz relative power was significantly reduced in rats in the 6-OHDA-lesioned group compared to that of rats in the control group (68.87 ± 2.29 vs. 80.82 ± 2.21; *p* < 0.01), while the relative power in rats in the DBS group was higher than that of rats in the 6-OHDA-lesioned group (76.46 ± 1.35 vs. 68.87 ± 2.29; *p* < 0.01). The 12–35 (23.07 ± 1.35 vs. 13.26 ± 1.40; *p* < 0.01) and 35–70 Hz (4.02 ± 0.54 vs. 2.30 ± 0.45; *p* < 0.01) relative powers were enhanced in rats in the 6-OHDA-lesioned group compared to those of rats in the control group, while both of these values were decreased in rats in the DBS group compared to those of rats in the 6-OHDA-lesioned group (12–35 Hz: (17.51 ± 1.27 vs. 23.07 ± 1.35; *p* < 0.01); 35–70 Hz: [2.51 ± 0.23 vs. 4.02 ± 0.54; *p* < 0.05)]. In general, compared with rats in the control group, the relative power of LFPs of the PF in rats in the 6-OHDA-lesioned group was significantly reduced in the 0.7–12 Hz band, and it was significantly increased in the 12–35 and 35–70 Hz bands. After GP-DBS treatment, the relative powers in the 0.7–12 and 35–70 Hz bands were reversed to the normal level and improved in the 12–35 Hz band. These changes were well reversed by GP-DBS treatment.

### Immunostaining result of tyrosine hydroxylase-positive neurons

**FIGURE 5:**
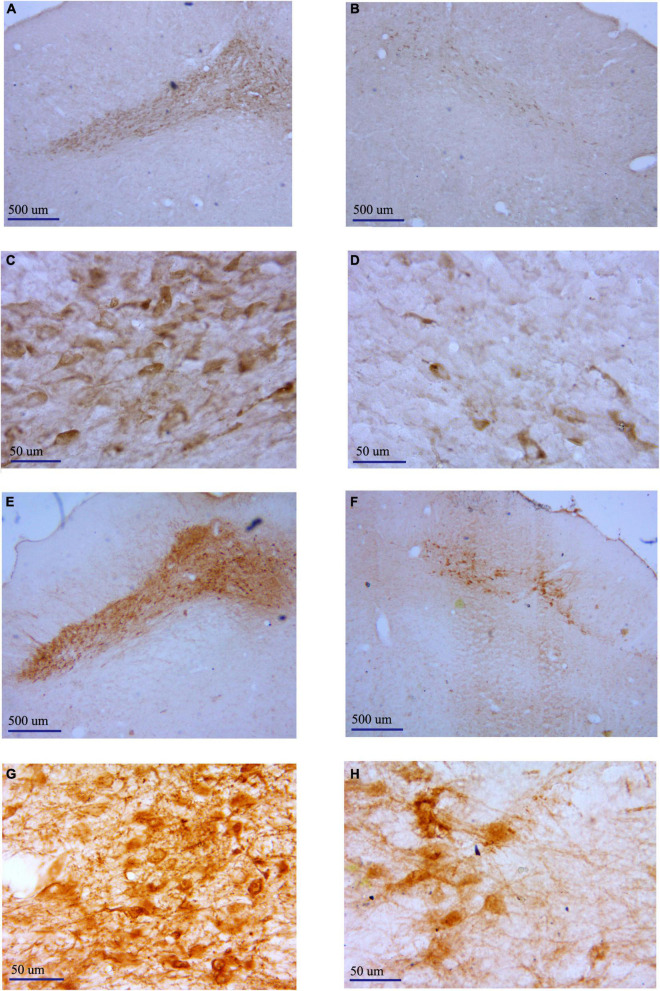
Representative pictures showing TH^+^ neurons in the SNc of different groups determined by immunostaining. **(A)** Non-lesioned side SNc of rats in the 6-OHDA-lesioned group (40×). **(B)** Lesioned side SNc of rats in the 6-OHDA-lesioned group (40×). **(C)** Non-lesioned side SNc of rats in the 6-OHDA-lesioned group (400×). **(D)** Lesioned side SNc of rats in the 6-OHDA-lesioned group (400×). **(E)** Non-lesioned side SNc of rats in the DBS group (40×). **(F)** Lesioned side SNc of rats in the DBS group (40×). **(G)** Non-lesioned side SNc of rats in the DBS group (400×). **(H)** Lesioned side SNc of rats in the DBS group (400×).

**FIGURE 6:**
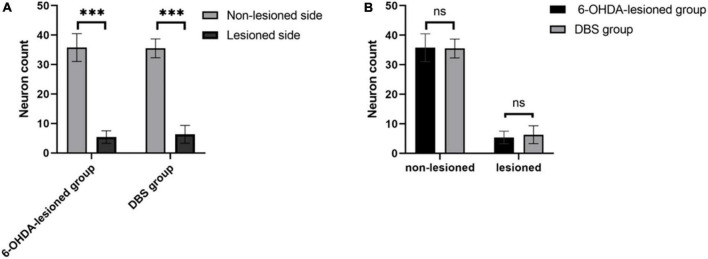
Counts of TH^+^ neurons in the SNc of rats in the 6-OHDA-lesioned group and the DBS group. **(A)** Comparison of the number of neurons in the non-lesioned and lesioned SNc in each group. **(B)** Comparison of the number of neurons in the non-lesioned and lesioned SNc between two groups. ***p < 0.001.

We observed TH immunostaining-positive neurons in SNc of rats in the 6-OHDA-lesioned group and the DBS group under the light microscope (Figures 5A–H). The neurons on the non-lesioned side of the SNc of rats in the two groups were distributed in strips. The neuronal cell body was full, densely distributed, and pyramidal or oval. The cytoplasm was brown-yellow after staining. Axons and dendrites of some neurons were clear. The distribution of neurons in the SNc on the lesioned side of rats in the two groups was generally shaped, but the number of neurons was small. We counted neurons in the SNc, including the non-lesioned and lesioned sides of the 6-OHDA-lesioned group and DBS group within a 6 × 10^4^ μm^2^ area under 400 × magnification (Figure 6). There was a significant difference in the number of SNc neurons between the non-lesioned side and the lesioned side in the 6-OHDA-lesioned group (35.78 ± 4.70 vs. 5.39 ± 2.12; *p* = 0); there was a significant difference in the number of SNc neurons between the non-lesioned side and the lesioned side in the DBS group (35.50 ± 3.20 vs. 6.33 ± 3.02; *p* = 0) (Figure 6A). There was no statistical difference in the number of neurons in the SNc on the non-lesioned side of the two groups of rats, and there was no statistical difference in the number of neurons on the lesioned side of the two groups of rats (Figure 6B).

## Discussion

In this study, a 6-OHDA-induced hemiparkinsonian rat model was established. The GP was chosen as the target for DBS treatment. The effects of GP-DBS treatment on the behavior of parkinsonian rats were evaluated *via* vibrissae-evoked forelimb placement and adjustment-step tests. Results from the vibrissae-evoked forelimb placement test showed that the ability of PD rats to raise their forelimbs to the table after DBS was significantly improved, reaching the level of the rats of the normal control group. Similar results were obtained in the adjustment-step test. The number of steps taken by the affected limb in PD rats after DBS also increased significantly but did not completely reach the normal level. The vibrissae-evoked forelimb placement and adjustment-step tests were used to evaluate the forelimb’s motor function on the rats’ injured side. Still, the initiating factors of their movement were different. The movement of the former is a reflex behavior induced by touching the whiskers. The latter is the active limb movement of the rat to move forward, so the evaluation results were not both restored to normal levels. Both behavioral tests showed that GP-DBS treatment remarkably improved the forelimb motor deficits of PD rats. These results were consistent with previous studies on GP-DBS in rats, monkeys, and PD patients (Vitek et al., 2004, 2012; Hahn et al., 2008). The behavioral results indicate that GP-DBS does have a certain therapeutic effect on PD symptoms. TH is a key enzyme in dopamine synthesis, and TH immunohistochemistry was used to label dopaminergic neurons (Shahnawaz Khan et al., 2012). The results of immunostaining TH indicated that GP-DBS could not prevent the damage of DA neurons in the SNc by 6-OHDA and could not regenerate the damaged DA neurons in the SNc. Most recent research on PD treatment with DBS has been concentrated on the GPi and STN. GPi and STN have been proven to have important value in treating PD (Wong et al., 2020; Au et al., 2021). GPi-DBS and STN-DBS can effectively improve the motor and non-motor symptoms of PD (Benabid et al., 2009; Fenoy and Simpson, 2014; Mueller et al., 2018). In contrast, the role and mechanism of GP-DBS have not been studied extensively. As an important part of the basal ganglia circuit, the GP directly projects its downstream nuclei, namely, the STN and GPi (Vitek et al., 2012). Some studies also suggested that GP-DBS may have potential application value in the treatment of PD (Daneshzand et al., 2017; Castillo et al., 2020). Yelnik et al. (2000) indeed found stimulation of the GP could improve akinesia of PD patients. Other studies have reported that GP-DBS can improve the motor symptoms of 1-methyl-4-phenyl-1, 2, 3, 6-tetrahydropyridine (MPTP)-treated monkeys, and physiological mechanisms revealed that GP-DBS could modulate the mean discharge rates and the temporal pattern of neuronal activity in the STN and GPi (Vitek et al., 2004, 2012). As a major basal ganglia hub, the GP has the most extensive efferent connections with basal ganglia structures and a unique direct projection to the frontal cortex. With the discovery of the importance of PF in basal ganglia-related movement disorders, especially in PD, PF has attracted increased attention (Ni et al., 2000; Villalba et al., 2014; Galvan et al., 2015). Through optogenetic technology, Lilascharoen et al. recently found numerous nerve fibers of the GP project to the PF (Yelnik et al., 2000). Our previous study found that the electrical activity of the PF nucleus in PD rats was changed under the quiet awake state, including the firing mode of a neuron and its local field potential (Wang et al., 2016). However, how the electrical activity of the PF nucleus changes after electrical stimulation remains unknown. This study observed electrophysiological changes in the PF after GP-DBS, including spike discharges and LFPs. To a certain extent, both the PF and the GP play important roles in the cortico-basal ganglia pathway. This result is of great significance for the study of the electrophysiological mechanism of GP-DBS in the treatment of PD.

In this study, compared with those in normal controls, the firing rates of PF neurons in 6-OHDA-lesioned rats did not change. The CV value of the two types of neurons in the PF both changed in the 6-OHDA-lesioned rats. The firing pattern of type I neurons in the PF of 6-OHDA-lesioned rats was more irregular than that of control rats, whereas the firing patterns of Type II neurons became more regular. The LFP of PF in PD rats also changed, with the relative power decreasing at 0.7–12 Hz and increasing at 12–35 and 35–70 Hz. GP-DBS treatment only reversed the enhanced CV value of type I neurons in the PF, which was used as an index for evaluating the discharge patterns. Moreover, the results demonstrated that GP-DBS treatment was associated with prominent variations in LFP power in the PF. Compared with those of parkinsonian rats, the relative powers of the GP-DBS treatment group decreased in the frequency ranges of 12–35 and 35–70 Hz but increased at 0.7–12 Hz, similar to the level observed in the control group. This result is consistent with our previous study (Wang et al., 2016). In recent years, an increasing number of researchers paid attention to the role of LFPs in PD (Angeli et al., 2015; Roy et al., 2018; Velisar et al., 2019; Au et al., 2021). This study showed that the modulatory influence of GP-DBS treatment on the altered neuronal activities of the PF is complex, not only in spikes but also in LFPs. At present, the exact mechanism of GP-DBS remains unclear. The study by Lilascharoen et al. (2021) showed that GP had a class of neurons directly projecting to the PF nucleus. Based on this result, we speculate that GP-DBS is likely to act on type I neurons of PF through such direct fiber projections, and the recovery of LFP of PF nucleus is also likely to be caused by the improvement of type I neurons. Though many anatomical and electrophysiological characteristics related to GP indicate that GP-DBS may mediate its effects through complex regulation of basal ganglia-thalamocortical networks (Yelnik et al., 2000; Vitek et al., 2012). Through our study, it can be determined that the change of PF nuclear activity is part of the electrophysiological mechanism of GP-DBS in the treatment of PD.

The PF is not a basal ganglia component, but it has complex fiber connections with the basic ganglia. The fibers of the PF project to the striatum. At the same time, the PF receives vital inputs from the GPi and SNr/STN (Parr-Brownlie et al., 2009; Smith et al., 2014). A recent study found that the PF also accepts projections from the GP (Lilascharoen et al., 2021). These findings supported the review that the PF has an important connection with the basal ganglia. A small node receiving DBS can make a vast network-wide difference that might profoundly improve several symptoms of PD and other movement disorders (Okun, 2012). We speculated that the PF participated in treating PD symptoms by GP-DBS with the basal ganglia; the recovery of the activity of the PF might be one of the important factors in affecting the PD-related pathway. To the best of our knowledge, the present study was the first to investigate how DBS affects the extracellular discharge activity in the PF in parkinsonian rats. Further studies are warranted to determine whether these changes in PF are caused by the direct action of the GP or the indirect action of other nuclei and how PF is involved in the alleviation of PD symptoms with the basal ganglia.

## Conclusion

In conclusion, our results suggested that GP-DBS improved the motor symptoms of PD model rats, so the GP nucleus is expected to become a new target for the treatment of PD. Moreover, GP-DBS also reversed the alteration of LFPs and partly improved the spike patterns in the PF of rats in the PD state. This result lays a foundation for the future study of the GP-DBS mechanism in the quiet awake state. Furthermore, the present results may contribute to a better understanding of the mechanism of DBS.

## Data availability statement

The original contributions presented in this study are included in the article/supplementary material, further inquiries can be directed to the corresponding author.

## Ethics statement

This animal study was reviewed and approved by the Animal Experimental Ethics Committee of Shandong Normal University.

## Author contributions

JX and TL designed the research. JX and TH performed the animal experiment and data analysis. JX, TL, and ZC wrote the manuscript. HZ, TC, CL, XF, and HS were responsible for the revision of the article. TH carried out the electrode production. All authors read and approved the final manuscript.

## Footnotes

1
http://www.nottingham.ac.uk/neuronal-networks/
